# Physiological correlates of cognitive load in laparoscopic surgery

**DOI:** 10.1038/s41598-020-69553-3

**Published:** 2020-07-31

**Authors:** Zohreh Zakeri, Neil Mansfield, Caroline Sunderland, Ahmet Omurtag

**Affiliations:** 1 Department of Engineering, School of Science and Technology, Nottingham Trent University, Clifton Lane, Nottingham, NG11 8NS UK; 2 Department of Sport Science, School of Science and Technology, Nottingham Trent University, Clifton Lane, Nottingham, NG11 8NS UK

**Keywords:** Biomedical engineering, Computational neuroscience

## Abstract

Laparoscopic surgery can be exhausting and frustrating, and the cognitive load experienced by surgeons may have a major impact on patient safety as well as healthcare economics. As cognitive load decreases with increasing proficiency, its robust assessment through physiological data can help to develop more effective training and certification procedures in this area. We measured data from 31 novices during laparoscopic exercises to extract features based on cardiac and ocular variables. These were compared with traditional behavioural and subjective measures in a dual-task setting. We found significant correlations between the features and the traditional measures. The subjective task difficulty, reaction time, and completion time were well predicted by the physiology features. Reaction times to randomly timed auditory stimuli were correlated with the mean of the heart rate ($r = - 0.29$) and heart rate variability ($r = 0.4$). Completion times were correlated with the physiologically predicted values with a correlation coefficient of 0.84. We found that the multi-modal set of physiology features was a better predictor than any individual feature and artificial neural networks performed better than linear regression. The physiological correlates studied in this paper, translated into technological products, could help develop standardised and more easily regulated frameworks for training and certification.

## Introduction

Laparoscopic surgery (LS) offers substantial clinical and economic benefits over open surgery, including decreased postoperative pain and better utilisation of hospital beds and antibiotics^1–3^. Consequently, LS is becoming increasingly routine in many surgical conditions. In the United States in 2013, surgeons performed cholecystectomy laparoscopically in 96% of cases^4^. The share of laparoscopy procedures within the total number of appendectomy and cholecystectomies in the United Kingdom in 2017 were respectively 74% and 92%, up from 41 and 84% in 2010^5^.

During LS, surgeons coordinate their hands, eyes, and long-shaft instruments in trocars inserted at narrow incisions, while mentally translating two-dimensional real-time video into the three-dimensional intracorporal setting. They continuously translate hand movements into the inverted movements of tool-tips and receive limited haptic feedback. The additional difficulties of performing LS relative to open surgery are widely acknowledged^6^. These highlight further unmet needs in the context of training and assessment, which would be greatly helped by tools that can provide new insights into skill development^7,8^.

In addition to technical skills based on visual and motor ability, surgeons generally use an array of non-technical skills that include mental readiness, cognitive flexibility, the ability to anticipate problems, team adaptation, safety awareness, situational awareness and communication style^9^. Intraoperative decisions are required when, for example, a surgeon faces an anatomic anomaly or patient decompensation. Rapidly making correct decisions and managing errors have been noted as critical components of surgical competence, which reduce the rates of patient mortality and complications^10,11^.

The exercise of non-technical skills may be impaired by excessive workload or stress. Under such circumstances a surgeon may be more easily distracted, entertain fewer alternatives, or persist with ineffective strategies^12^. Some of these scenarios have been discussed in the context of LS^13^. If the primary technical task is difficult, for example, because the surgeon is continually mentally adjusting for mismatches between the endoscope and the instruments on the monitor^14^, they will have fewer resources available to devote to anticipating the next steps or detecting a potential problem before it becomes an emergency. Focussed on completing a suture, a surgeon may miss a crucial alarm; an instance of inattentional blindness/deafness^15,16^. Or a trainee may reach a performance plateau where additional training does not improve overt patterns but continues to reduce the perceptual-motor and cognitive demands of the task^17^. These considerations suggest that performance based metrics alone may not suffice to reveal the trainees' actual state of readiness.

Although not as directly observable or well-defined as performance itself, cognitive load has nonetheless been operationalised in multiple ways. An established technique involves introducing a secondary task to be done concurrently, and observing the extent to which it affects the performance of the primary task^18^. Secondary task methods have been used to measure the cognitive load associated with LS^19,20^. A common technique is to employ a secondary task involving the operator's ability to respond to an environmental stimulus such as a visual or auditory change. In this case the reaction time (the duration between stimulus onset and the subject's response to it) and detection rate (e.g. the fraction of times the stimulus is responded to) are taken as the relevant performance measures^21^.

There are, in addition, numerous survey based approaches that obtain information from the self-reporting of the subjects' experience. One of the most widely used is the questionnaire known as NASA-TLX^22–24^. The amount of effort thus measured can be assumed to indicate the operator's residual capacity available for dealing with unexpected events and for planning and strategy^25,26^. Secondary task and subjective ratings were concurrently used in measuring the cognitive load of novices in the early phases of LS skills training, with subjects monitoring a patient's vital signs (secondary task) while tying surgical knots^27^. These traditional measures face some limitations, as they may influence or interrupt what they are trying to monitor.

As alternatives to secondary task and subjective methods, physiological measures are available for tracking operator states, often in the form of wearable devices. They are gaining in popularity as progress in sensor technology makes this approach less intrusive and capable of delivering continuous, multi-modal information. Physiological responses are tightly coupled to the experienced load during task performance, and cardiac and ocular measures are among the most informative physiological indicators^12,28,29^.

The heart rate (HR) and heart rate variability (HRV) are autonomic response markers which can quantify stress and cognitive load in the surgical context^30^. The changes in intraoperative HR tend to be larger than can ascribed to physical and musculoskeletal demand ^31^. Accordingly, HR has been used as an indicator of acute stress in surgeons^32,33^.

Under resting conditions HR typically oscillates, increasing/decreasing during inspiration/expiration, in what is known as the respiratory sinus arrhythmia (RSA)^34^. The abatement of RSA by the sympathetic system correlates with forms of arousal and cognitive load^35^. HRV is used to describe such changes in RSA that are readily derivable from the electrocardiogram. HRV may detect autonomic changes even when measures of HR remain constant. It was found to decrease during the surgical decision making steps^36^, could detect the cognitive load associated with LS^37^, and helped identify the segments of coronary bypass surgery that were differentially stressful for attending and resident surgeons^38^.

Healthy, resting subjects spontaneously blink their eyes approximately 14 times per minute. These endogenous eye blinks occur more frequently than needed for maintaining the corneal tear film, which may be their primary function^39^. Endogenous blinks (in contrast with the protective reflex and voluntary ones) are driven in part by dopaminergic processes originating from basal ganglia, and their frequency and duration reflect mental states^40–42^. Since blinks momentarily block vision, it is plausible that they would be suppressed when expecting important visual input. Evidence supports this view^43^, but also suggests that the blink rate (BR) decreases with increasing attention and task engagement regardless of stimulus modality^44–48^.

The goal of the present study was to investigate the ability of physiological variables to track the level of effort associated with LS training tasks. Our study focussed on the major non-cerebral responses described by cardiac and ocular variables, HR, HRV and BR. These were derived from concurrent near-infrared spectroscopy (fNIRS) and electroencephalography (EEG) recorded from novice subjects. As they performed tasks on a LS trainer box, the subjects responded to randomly timed auditory stimuli by pressing a pedal. They reported their experience by filling out a questionnaire after each session. We postulated that the physiological metrics would track the overall effort as indexed by the traditional secondary task and subjective methods, and sought to quantify their ability to do so through statistical and machine learning techniques^49^.

At present, there is limited research on the extent of the agreement among these distinct measures of effort and no studies to our knowledge in the context of LS training. In our study, varying levels of effort were generated by differences in subject aptitude and task difficulty, as well as by learning and time on task effects. We believe physiological responses which track their effects are more direct than behavioural and verbal ones, and are more easily measured. Thus, a better understanding of the physiological expression of skill is of potential value for improving training and assessment in LS.

## Results

**Figure 1:**
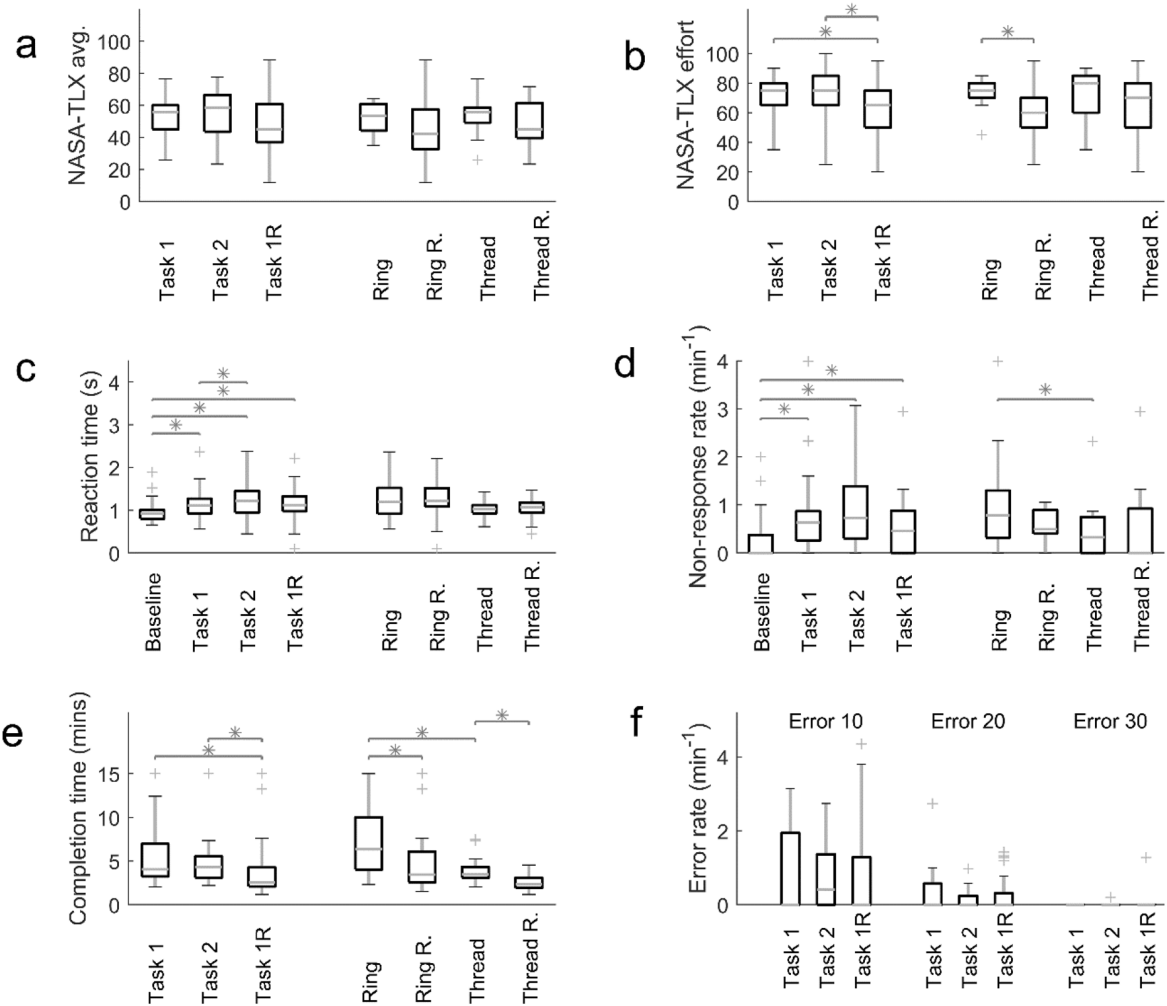
Experience and performance metrics grouped by type of experimental episode (three boxes on the left of each subplot) and by task type, namely Ring Transfer, Ring Transfer Repeated, Threading and Threading Repeated (four boxes on the right). (**a**) NASA-TLX Average score. (**b**) NASA-TLX Effort score. (**c**) Reaction time. (**d**) Non-response rate. (**e**) Task completion time. (**f**) The rates of errors during the Ring Transfer task. (*p < 0.05).

**Figure 2:**
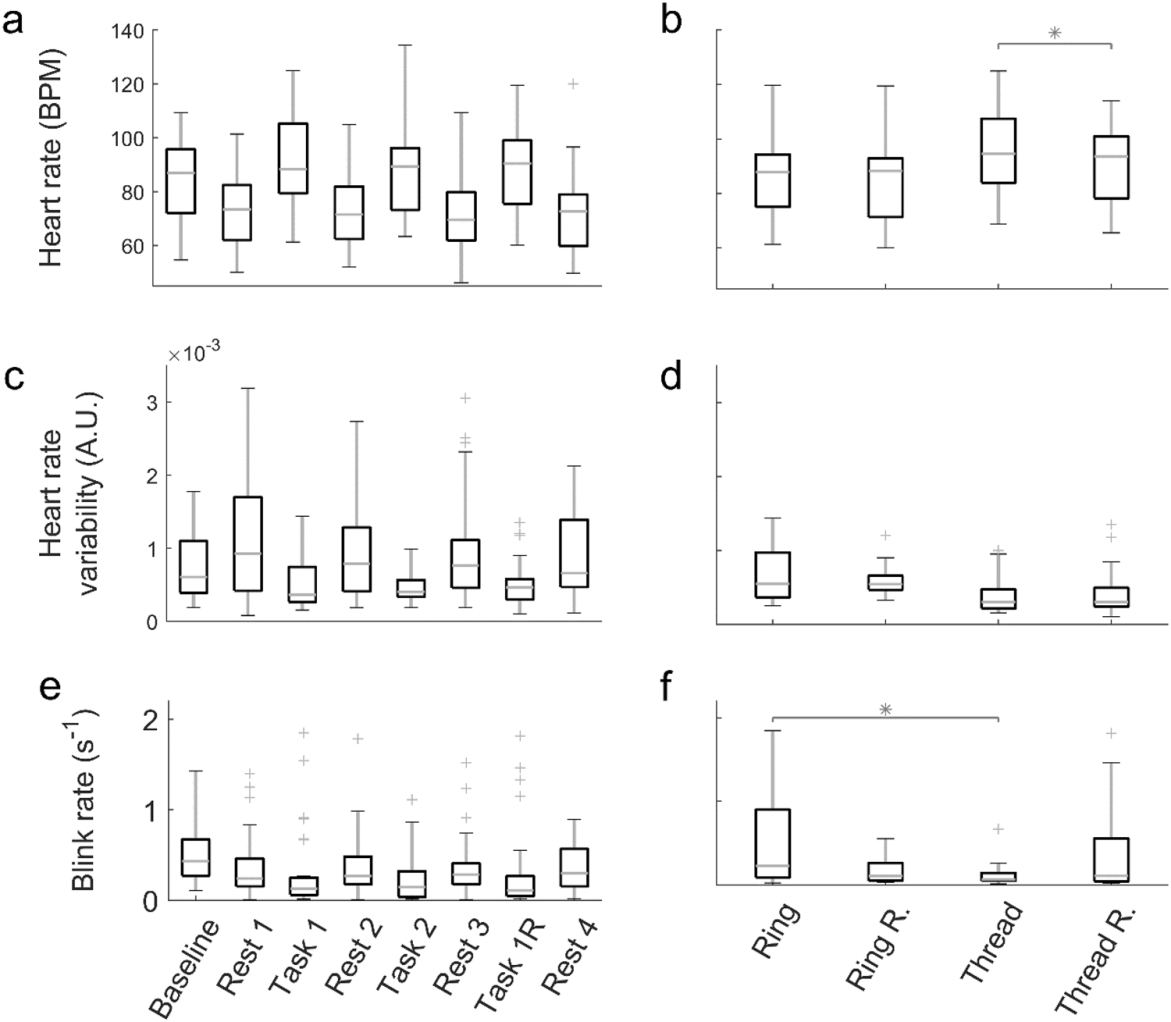
Physiological metrics, heart rate (HR) and heart rate variability (HRV) extracted from fNIRS signals and blink rate (BR) from EEG. (**a**) HR grouped by experimental episode. (**b**) HR grouped by task type and task-specific repetition. (**c**) HRV grouped by experimental episode. (**d**) HRV grouped by task type and task-specific repetition. (**e**) BR grouped by experimental episode. (**f**) BR grouped by task type and task-specific repetition. Significant differences were not marked in **(a)**, **(c)** and **(e)** in order to avoid clutter. (*p < 0.05).

**Figure 3:**
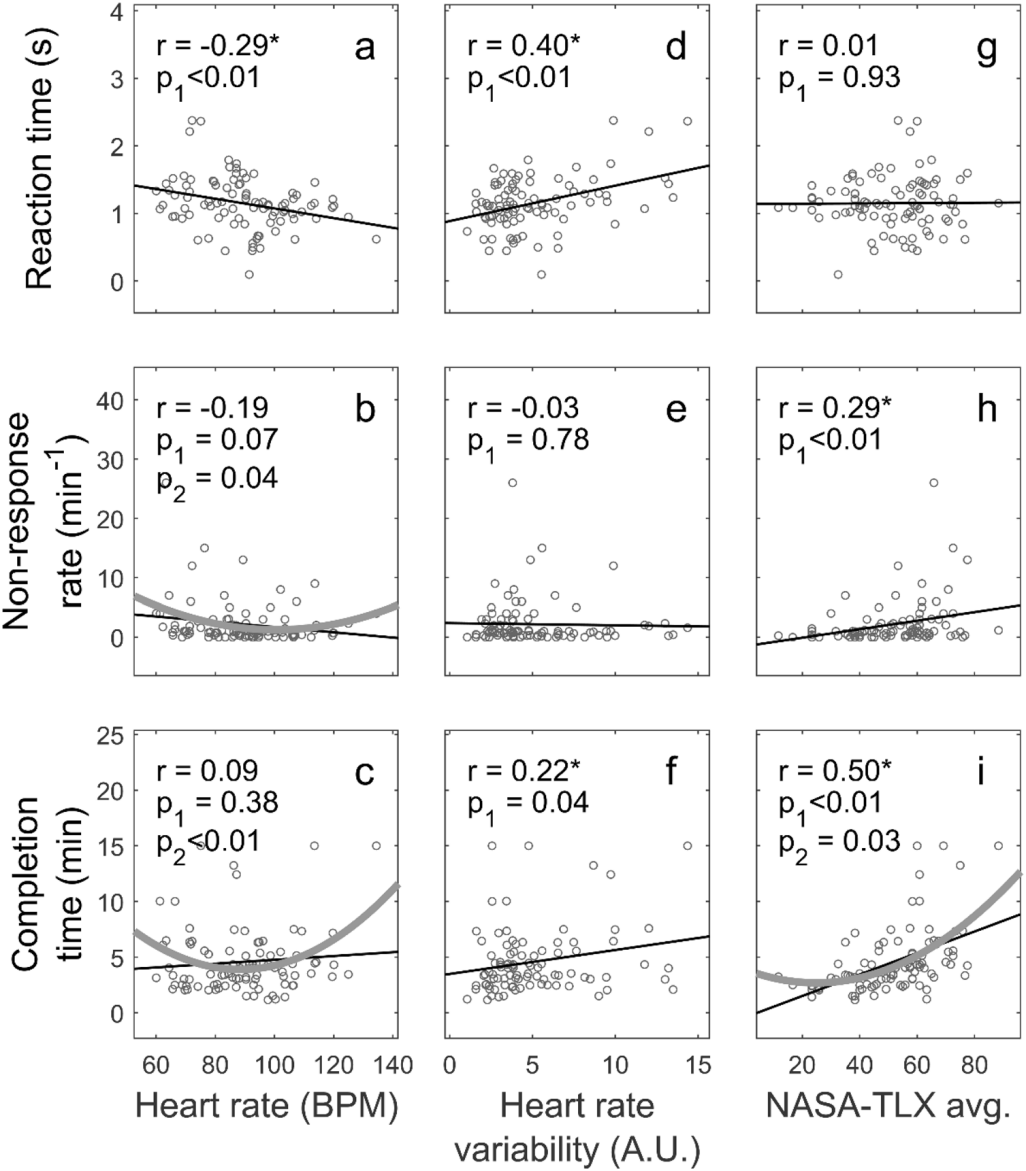
Values of the individual predictor features and targets. Linear fits to the data are shown. The Pearson correlation, $r$, and the p-value of linear regression (black line) are shown. The quadratic fit (thick grey curve) and its p-value $p_{2}$ are shown only if $p_{2} < 0.05$.

**Figure 4:**
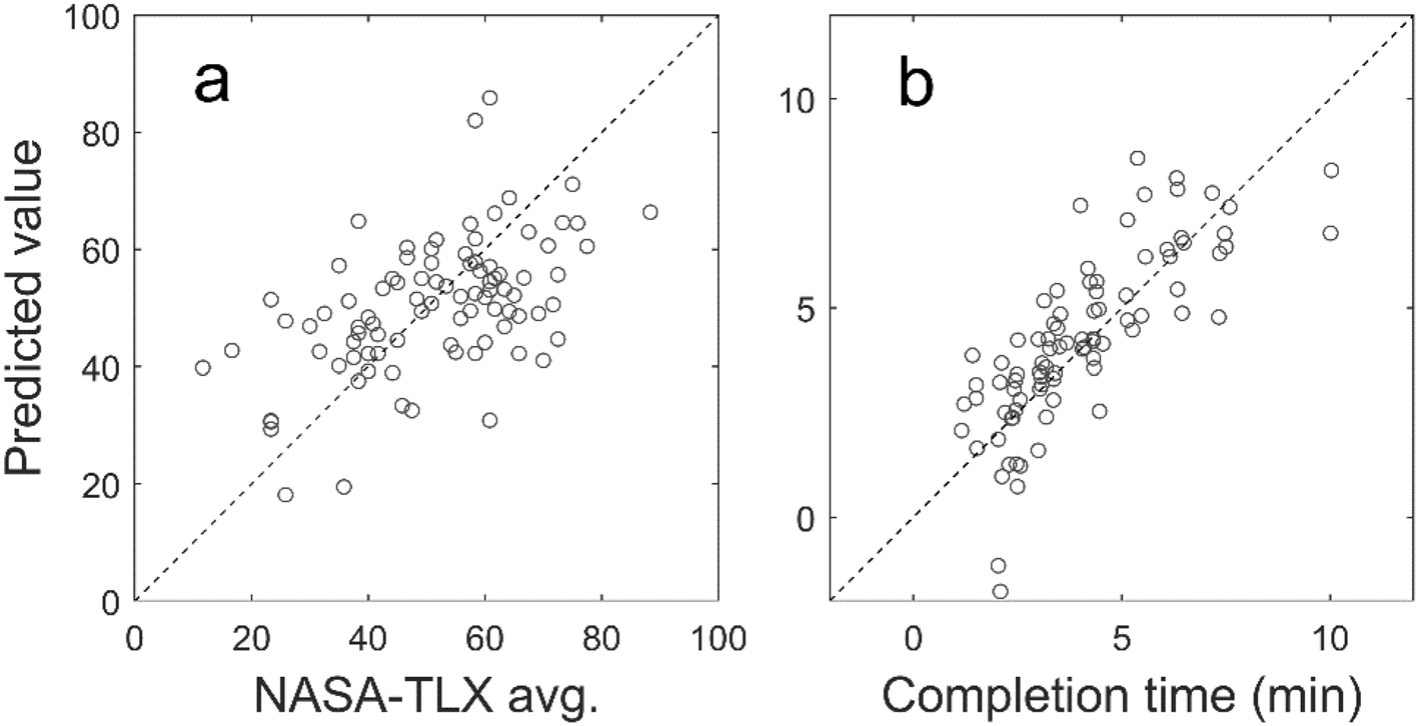
Predictions of the artificial neural network plotted against the actual values (x-axis) for selected cases. (**a**) NASA-TLX Average score with $R_{CV} = 0.60$. (**b**) Completion Time with $R_{CV} = 0.84$. The 45-degree diagonal line corresponds to perfect prediction.

**Figure 5:**
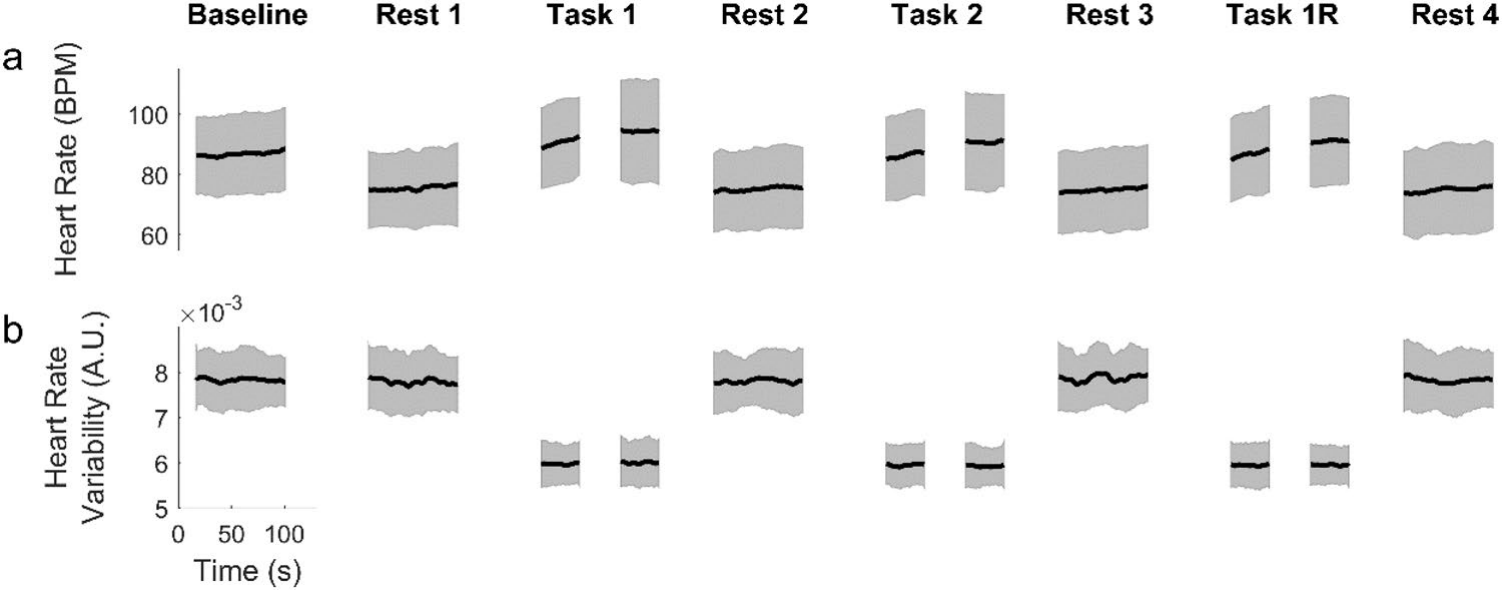
Heart rate (**a**) and heart rate variability (**b**) extracted from NIRS signals for the experimental episodes, and smoothed by a 10 s moving window. The black curves represent subject average and the shaded regions are the standard deviation of subject variability. For the task episodes, 40 s segments at the beginning and end are shown.

**Figure 6:**
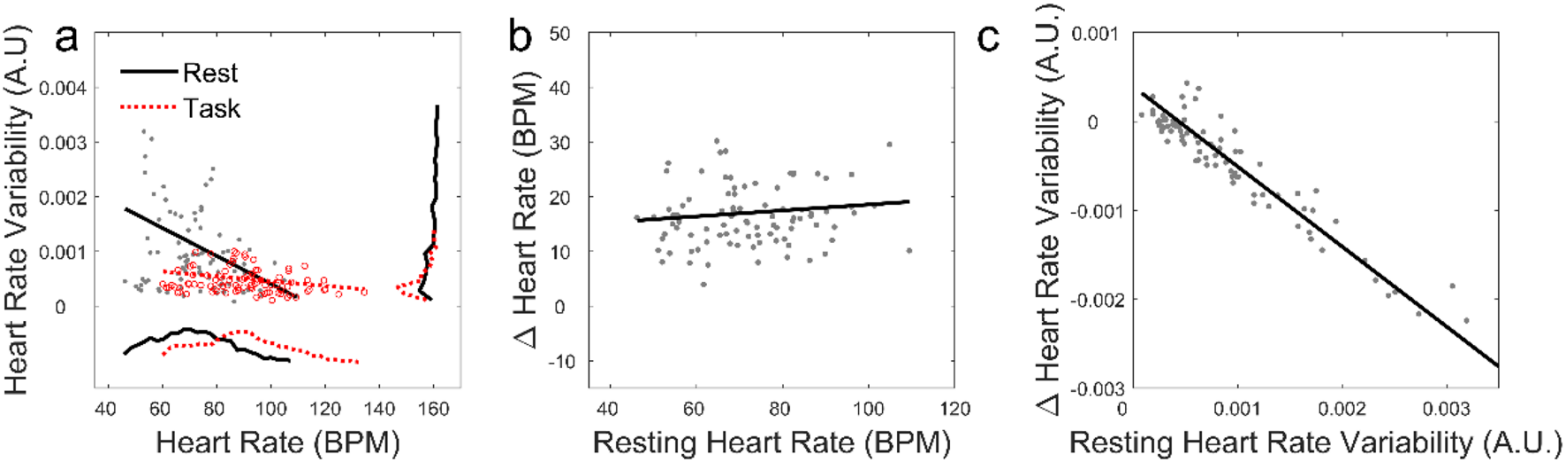
Relationships among the heart rate (HR) and heart rate variability (HRV) for Rest and Task. (**a**) HR and HRV in resting (black) and task performance (red). Black and red dots respectively represent individual subjects in the resting and task blocks. The linear fits to the data are shown as the black and red straight lines. The Pearson correlations are $r = - 0.32$(resting) and $r = - 0.23$ (task). The insets at the bottom and on the right illustrate the marginal probability distributions, with x-axes matched to the main figure. (**b**) Resting HR and the task related change in HR relative to resting ($r = 0.11$). (**c**) Resting HRV and the task related change in HRV relative to resting ($r = - 0.96$).

In Figs. 1 and 2 we present the descriptive statistics of the data collected. That is followed by the prediction of cognitive load from physiological variables. Figures 3 and 4 drill down into the details of the some of the associations revealed in Table 1. Further results elicited by analysis of HR and HRV are displayed in Figs. 5 and 6.

**Table 1:** Prediction performance for the task episodes. All data from the tasks (T1, T2, T1R) were pooled.

Target	Predictor										
	Heart rate ($r$)				Heart rate variability ($r$)				Blink rate ($r$)	LR ($R_{adj}$)	ANN ($R_{CV}$)
	Mean	Std dev	Max	Min	Mean	Std dev	Max	Min	Mean		
NASA-TLX avg	0.18	0.19	0.2	− 0.08	− 0.14	0.04	0.05	− 0.33*	0	0.48*	0.60*
Reaction time	− 0.29*	− 0.1	− 0.17	0	0.40*	0.31*	0.41*	0.30*	− 0.21*	0.47*	0.56*
Non-response rate	− **0.19**	− 0.04	− 0.05	− 0.11	− **0.03**	0.09	0.06	− 0.11	0.03	0	0.36
Completion time	**0.09**	0.02	− 0.03	0.04	0.22*	0.35*	0.47*	− 0.11	0	0.77*	0.84*
Error rate (type 10)	− 0.19	− 0.15	− 0.1	0.08	0.2	0.17	0.26*	0.04	0.11	0.44*	0.26

The last two columns on the right show the values of $R_{adj}$ and $R_{CV}$ for the performance of regression and artificial neural network, respectively, using the full set of features. Individual features of heart rate, heart rate variability and blink rate are shown in separate columns. For each feature the table shows the Pearson correlation ($r$) between the feature and target. The values in bold were chosen as examples to be examined further. (*p < 0.05).

The experience related and behavioural variables are given in Fig. 1. The first row is the subjects' experience as reported in their NASA-TLX scores. The NASA-TLX questionnaire asked subjects to score the task along the dimensions Mental, Physical, Temporal, Performance, Effort and Frustration, on a scale from 0 to 100 in increments of 5. The average of these dimensions is shown in Fig. 1a. The overall shifts in scores were evident and consistent with expectations. Similar shifts in the average score were present in all the individual dimensions; we show only Effort scores in Fig. 1b. Particularly clear were the decreases which are observed when tasks are repeated. In Fig. 1b the Effort scores are selected for display because most of the repetition effects were statistically significant. Performance scores (not shown) also contained changes that were statistically significant. There did not appear to be a significant difference between the experiences of the Ring Transfer and Threading tasks.

The secondary-task based measures had a greater number of statistically significant between-group shifts as shown in the second row of the figure. The reaction time (Fig. 1c) was shortest during the Baseline, as expected, since the pedal press task was then being performed by itself. The reaction time tended to increase (both its median and range) during task performance, and visibly dropped when the first task was repeated. The non-response rate (Fig. 1d) showed a similar pattern of increase followed by a decrease related to repetition. The non-response rate was a significantly greater for Ring Transfer than for the Threading task. The non-response rate was the rate at which a subject failed to respond to the stimulus before the next stimulus onset.

The primary task's completion time (Fig. 1e) also showed a strong repetition effect. The completion of Threading appeared to be significantly quicker than that of the Ring Transfer. The rates of error did not display strong trends (Fig. 1f) although the increase in the median of type 10 Error in going from the first to the second task appeared to mirror the experience and secondary task variables. Error type 10 was dropping a ring on the ring stack board; type 20 was dropping it outside the board; and type 30 was dropping it outside the trainer box.

The physiological variables are shown in Fig. 2. The left column of the figure shows robust differences between the Rest and Task episodes for HR, HRV and BR. In fact, each Rest episode was statistically significantly different from each Task episode; therefore, the usual asterisks were omitted from the left column to avoid cluttering. In addition, the Baseline values for HR and HRV were situated about halfway between Rest and Task, consistent with the expectation that performing only one task is easier than performing two at the same time. In the case of HR, the difference between Task 1 and its repetition was significant, while this was not the case for HRV or BR. Figure 2a shows a significant difference in HR between the first and repeat performances of Threading, while Fig. 2f indicates that BR was far lower in Threading than in Ring Transfer.

In the boxes in Figs. 1 and 2, the central mark indicates the median, and the bottom and top edges of the box are the 25th and 75th percentiles. The whiskers extend to the most extreme data points not considered outliers. Outlier data are shown as the ' + ' signs. A large number of outliers (e.g. Fig. 2e) suggests a non-normal distribution. Outliers were defined as points greater than $q_{3} + w(q_{3} - q_{1} )$ or less than $q_{1} + w(q_{3} - q_{1} )$, where $q_{k}$ is the $k^{th}$ quartile and $w = 2$.

Having found task-dependent patterns in the subjective, behavioural and physiological data with different degrees of inter-subject variability, we next proceeded to the central concern of this study, namely the extent to which the physiological variables could predict experience and behaviour that are traditionally used for measuring effort. Table 1 shows how HR, HRV and BR derived variables (predictors) related to the traditional variables (targets), individually as well as collectively via machine learning approaches. The Table shows the Pearson correlation ($r$) for individual features. It also shows the adjusted-R and cross-validated-R, explained in the "Methods" and Supplementary Information sections, for assessing the performance of the linear regression (LR) and artificial neural network (ANN).

Regarding the individual features in Table 1, the Pearson correlations show that HR's mean and standard deviation tended to increase with the experienced difficulty as measured by the NASA-TLX score. Without reaching statistical significance both were individually positively correlated with it. Mean HR was negatively correlated with the Reaction Time and Non-response Rate. Higher HR generally implied quicker and more accurate response in the secondary task and fewer errors in the primary task. HRV appeared to be an even better predictor. The minimum value of HRV was significantly negatively correlated with the NASA-TLX score. Many HRV features were significantly positively correlated with the Reaction Time, Completion Time, and Error Rate. This indicated that higher HRV (which is associated with respiratory modulation of HR and relaxation) degraded performance. The BR, although not as predictive as the others, was significantly negatively correlated with the Reaction Time.

Note that many of the correlation values in Table 1 may be considered as low or negligible^50^. Those predictors do not have high degree of linear dependence on their target. Note, however, that the value the Pearson coefficient is distinct from its statistical significance. (The latter is indicated by an asterisk in the table.) We used linear correlation only as a baseline to compare with the performance of groups of physiological metrics that had predictive utility via machine learning (the right column of the table).

With regard to the LR and ANN which used all available features (the last two columns on the right of Table 1), the results indicated that the full set of features collectively outperformed any individual feature in predicting subject experience and performance. In almost all rows, the table shows that $R_{adj}$ and $R_{CV}$ were greater than the absolute values of the individual Pearson correlations. Furthermore, ANN outperformed LR in almost all cases, except in predicting the Error Rate which was the unique case with $R_{CV} < R_{adj}$.

Having shown how physiological variables were related to experience and behaviour, we looked more closely at the relationships that were quantified in Table 1. We examined the scatter plot of the predictor and target data points in Fig. 3. The figure shows in subplots a-f the cases shown in bold for individual features of Table 1. In subplots a-c, the dependences of the Reaction Time, Non-response Rate and Completion Time on HR are described. In subplots d-f, the same targets are shown plotted against HRV. Finally, since the NASA-TLX score was traditionally used as a measure of stress (as HR and HRV), we also show in subplots g-i the same targets plotted against the NASA-TLX scores. In addition to the linear regression shown as the thin black line in each subplot, we also computed a best fit by including the square of the predictor. Only if the fit was statistically significant, we included this quadratic regression as a thick grey curve in the plot. When the quadratic regression was significant (subplots b, c and i) the best fits were U-shaped curves, suggesting that there was a performance optimum.

Continuing with the scrutiny of the results in Table 1, we show a scatter plot of the actual and predicted values of two variables in Fig. 4. These correspond to the predictions by ANN shown in bold in the last column on the right of Table 1. Completion Time (Fig. 4b) was the most accurately predicted target, with $R_{CV} = 0.84$, while the NASA-TLX score (A) was the second best, with $R_{CV} = 0.60$.

We described in Fig. 2 the episode averages of the HR and HRV. However the heart beats approximately once per second, which is sufficiently frequent to allow the temporal variations of HR and HRV within episodes to be observed. These variations are illustrated in Fig. 5 that shows the time courses of the subject mean (black curves) and inter-subject variability (shaded regions). For the task episodes only the beginning and end segments are shown since the task duration was different for each subject. The figure suggests that during task performance the HR and HRV reach values that are anti-correlated and, as such, potentially contain comparable amounts of information about effort. However, the intra-episode time courses reveals a significant difference between these two measures. While the HR climbs gradually up to its maximum level within the first minute or so, the HRV attains its task-related minimum without any visible delay.

Figure 5 showed differences in the time-courses of HR and HRV. However, there are additional differences in how the change in these metrics depend on their starting point. This is partly revealed in Fig. 6a, a scatter plot of HR v HRV during the rest (black dots) and task (red dots) periods. The linear regression lines to the data are shown in the corresponding colours. The distribution of HR and HRV are also shown at the bottom and right. Task performance causes the HR distribution shift to the right as a whole, while the distribution of HRV changes shape and becomes more concentrated at small values. The reasons for these differences become evident when we consider how the change in HR (in Fig. 6b) and HRV (in Fig. 6c) depended on the subjects resting values. The best fit in Fig. 6B is almost flat suggesting that the shift in HR does not depend on its starting point, while it is sloping down in in Fig. 6c suggesting that the shifts in HRV are strongly negatively correlated to their starting points. This indicates that only the HRV of those subjects that have high resting HRV are affected by task performance.

## Discussion

This paper showed that the cognitive load on novice trainees during LS training has physiological correlates. We showed that physiological indicators of cognitive load predict experience and performance. As physiological metrics, we calculated from N = 31 subjects the heart rate, heart rate variability and blink rate. We measured their subjective experience by the NASA-TLX reports and their performance in a dual-task setting. We used regression and artificial neural networks to discover relationships among the variables. Our hypothesis was that the residual capacity revealed traditionally by reports and other measures such as reaction time would be reflected in their physiology. The motivation was to develop new metrics to guide training so that surgeons train not only to become good at their primary technical tasks, but to have sufficient capacity for planning and for unexpected events. Although some combination of HR, HRV and BR as well as NASA-TLX and dual-task performance have been used in the past to investigate workload in surgery, we are not aware of other studies which brought all of these variables and machine learning together, within the experimental setting of laparoscopy training.

We found that changes in physiology, experience and performance depended on whether subjects were resting, performing the secondary task only (baseline) or both tasks (Figs. 1 and 2). Many individual physiological features had some predictive power (Table 1). From least to most effective were BR, HR and HRV features. The superiority of HRV came at the cost of the additional computations that were required. ANN were more effective than LR in predicting experience and performance from physiology, suggesting that the relationships were non-linear. However, the accuracy advantage of ANN also came at the cost of additional CPU time (1.8 s per round of cross-validation, as compared to 0.07 s for LR). In addition, the full set of features was collectively more predictive than any individual feature. Such synergistic operation of physiological indicators was noticed previously in measuring the mental workload of nuclear power plant operators ^45^. HRV had the additional advantage of responding to task difficulty within the first few seconds, much faster than HR (Fig. 5). The regression-to-the-mean which we observed in HRV (Fig. 6) has been shown in a mental arithmetic task^51^. To interpret this in another way, note that HRV has a lower bound near zero and, and HRV that is already low at baseline consequently has little room to further decrease under task conditions. Here we have explored potential uses of HRV as a biomarker; the underlying mechanisms of HRV have been extensively probed in other studies^52^ .

In Table 1 both the predictor and target values were selected from the task episodes. We have repeated the calculations in two different ways. First, we repeated them by taking the predictor variables not from the task episodes but from the first rest episode. This was done in order to assess the amount of information carried by the subject's resting physiology about their experience and performance during task. Results showed that in this case all values in the table (except Reaction Time) became smaller and statistically insignificant. The predictability of the Reaction Time from the HR mean, HRV minimum, and LR, remained approximately the same. Second, we took the predictor variables as the change from rest to task. In this case, the predictability measures improved compared to the resting predictors. For example, many values in the last two columns on the right of the table remained significant. Nonetheless, all the values were lower, suggesting that the task physiology by itself was a better predictor than the physiology of task relative to rest, or rest alone. This is consistent with previous finding on the physiological correlates of cognitive performance and attention in non-surgery contexts^29,53^.

Our results suggested that stress helps improve primary task performance, indexed by Completion Time, up to a point, after which it degrades performance (Fig. 3). Here, stress was quantified by HR and by NASA-TLX score^12^. This may be the first evidence (to our knowledge) for the Yerkes-Dodson law in the context of surgery training. The law postulates an inverted U-shaped relationship between unspecific arousal and cognitive performance^54^. Given that the stress-performance curves may differ among surgeons, objectively evaluating them may improve training as well as optimise intra-operative conditions for performance.

The physiological metrics we have found correlated well with subjective and dual-task measures, but not well enough to replace them. We believe physiological correlates may contribute to surgery training not necessarily by eliminating other metrics but by providing additional, sometimes more practical, tools; especially if they are encapsulated in wearable systems. We have only considered cardiac and blink variables, but the number of relevant physiological metrics could be vast, given the complexity of the physiological processes, which underlie skill. Although insight into such underlying mechanisms is still in progress, the metrics can still be used for prediction with the help of machine learning. Furthermore, our results do not depend on the specific modalities (fNIRS and EEG) which were used to extract this information. Their detection could be based on different principles, for example, blinks can be extracted unobtrusively from video.

This study was motivated by the view that surgical skill is an important factor that impacts patient outcomes. It has been shown that this impact can be clearly distinguished from that of other factors that interact with it, such as the technology used, patient risk, and the overall quality of care. (An example of such interactions is that difficult cases may be taken up by more highly skilled providers, thereby selectively lowering their success rate.) Recent studies that controlled for these confounds have revealed that surgeons in the top quartile of skill level had significantly better outcomes than those in the bottom quartile^55,56^. We were also driven by the view that wearable sensors stand to play an important role in conveniently tracking the physiological correlates of skill. However future research needs to focus more on the effects of the sensors themselves on stress as well as on the normal range of physiological responses.

The types of physiological metrics investigated in this study may benefit skills training in robot-assisted surgery^57,58^, in the wider field of human–robot interaction^59^ and in sports^60^. We believe that the need for training human surgeons is not likely to diminish in the near future, as surgery is not one of the occupations at risk of being automated^61^ .

Our study had some limitations. These included: (i) Using ANNs without optimisation of parameters; (ii) Not implementing subject-specific calibration; (iii) Not controlling for levels of motivation or psychomotor aptitude among the novice subjects, although these variables may have accounted for the distribution in our results; (iv) Recruiting only novice subjects, while different known levels of expertise among the subjects would have provided additional insights; (v) Lack of training interventions accompanied by longitudinal tracking. We hope and expect that these limitations will provide opportunities for further study.

Laparoscopy has been spreading, owing to its clear advantages in safety and cost, short hospital stays and rapid return to work. These advantages can translate into significant economic benefits, reducing national^62^ and global^63,64^ disparities in surgical care. The physiological correlates of surgical performance studied in this paper, translated into technological products, could shrink the learning curves and help develop standardised and more easily regulated frameworks for training and certification^65,66^.

## Methods

### Participants

Thirty-eight healthy adult volunteers without any prior experience in laparoscopic surgery participated in one main trial to perform pre-determined basic laparoscopic tasks on a laparoscopic simulator. We excluded seven participants; one could not enrol in the experiment due to participant’s hairstyle, that made it impossible to fit the cap and record EEG; for four participants, technical problems prohibited recording; two were excluded from the main analysis due to a problem with the time stamps routine. Therefore, complete data sets from 31 participants were used in this study (17 females and 21 males, age: 21.61 ± 2.12 years). All participants provided their written informed consent prior to the study commencing and received gift vouchers for participating after the experiment was completed. Participants had normal or corrected to-normal vision. The Ethical Committee of the College of Science and Technology at the Nottingham Trent University approved the study and all research was performed in accordance with relevant guidelines.

### Experimental procedure

The experiment was conducted in a training lab that consists of a LS trainer box by Inovus Surgical Solutions (The Pyxus laparoscopic box trainer by Inovus Medical—43 cm × 33 cm × 31 cm) and a 21-inch monitor. Each trial took about an hour including total time spent by participants to perform the tasks and setting up the system and devices. At the beginning of the trial, participants received instructions about the session via a training video followed by a few minutes of hands-on introduction for orientation with the surgical equipment (Maryland Grasper and Needle Holder, Inovus Medical, St Helens, UK) until they became competent before commencing on the training. The LS trainer box was equipped with a centrally mounted camera and a light source with entry ports of the instruments separated by 13.5 cm. The training bases (14 cm × 10 cm) were placed in the LS trainer box and centred in the camera’s field of view. Laparoscopic video was projected onto the monitor that was in the direct view of the participant.

The experiment started by performing the secondary task alone for two minutes, which was considered as a baseline (Fig. 1). For the rest of the experiment, participants performed primary and secondary tasks simultaneously. The primary tasks included two Fundamentals of Laparoscopic Surgery tasks, Ring Transfer and Threading, in alternating sequence (Task1 and Task 2) followed by repetition of the first task (Task 1 Repeated). Fingertip blood samples were taken at baseline and immediately after completion of all three LS tasks to determine the serum cortisol and brain-derived neurotropic factor (BDNF) concentrations. After each blood sample, participants filled in the NASA-TLX questionnaire for each LS task. A rest period of 2 min was taken after the initial secondary task performed alone and after each blood sample, prior to performing the next task. Blood sample analysis is not included in this paper.

Participants were instructed to stand in front of the LS trainer box during the performance of Laparoscopic and secondary tasks. The laparoscopic tasks completion time was recorded, with a maximum time on task of 15 min after which the participants were told to stop.

The Ring Transfer task involved grasping, lifting and relocating rings from one rod to another using both surgical instruments and was performed on a ring stack base (Inovus Medical, St Helens, UK) (Supplementary Fig. S1). Four rods were selected and labelled A, B, C and D, at the left-hand bottom, top left-hand, top right-hand and bottom right-hand corners on the ring stack base respectively. Four rings were initially put over rod A at the beginning of the trial. The procedure includes picking up a ring from rod A and placing it onto rod B with the left-hand only. After transferring all four rings to rod B, participants used their left-hand only to grasp and lift up each ring, pass it to the right-hand and place it on rod C. The procedure was completed by moving the rings individually from rod C to D using the right-hand only. If any rings were dropped during the task completion, they were placed back on the rod they were taken from by an experimenter, and participants were allowed to continue the trial. The Threading task consisted of passing a piece of string through the holes in a pre-determined order. The holes were labelled 1–7 in a zigzag pattern on the Threading base (Supplementary Fig. S2). Participants could use both surgical tools, however, no restriction was made on the use of right, left or both hands. Timing began upon first grasp of the string that was initially placed on the right-hand side of the Treading base.

In order to simulate the potential distractions or disruptions (e.g. auditory alarms) that may arise in a realistic setting, an auditory task was added to the experiment. The secondary task, as a disruptive stimulus, provided an additional measure of the cognitive load on the participants in terms of reaction time and non-response rate. Participants had to respond to a series of beeps as quickly as possible by pressing down on a foot pedal. The beeps were generated with random frequencies (ranged from 1,000 to 2000 Hz), intervals (ranged from 3,000 to 10,000 Hz) and durations (ranged from 500 to 1,000 ms).

### Data collection

Participants’ brain electrical activities were collected using a wireless data acquisition system by TMSi Mobita^67^ with potentially up to 32 gel-based EEG channels, following the international 10–20 system of electrode placement. The EEG signal was collected at 2000 Hz using 19 electrodes out of 32 with average reference, and a ground electrode integrated in a wristband that was attached to the participant’s wrist. The fNIRS data were acquired at 10 Hz using Artinis Octamon continuous wave system^68^. The eight fNIRS channels (transmitter–receiver pairs with separations in the range 20–30 mm) covered a region approximately between FP1-F3-F7 on the left and its symmetric counterpart on the right. Hence they sampled parts of ventro- and dorsomedial prefrontal and orbitofrontal cortices^69^. These were combined with EEG electrodes into a single head-cap.

EEG and fNIRS signals were synchronised using PortaSync from Artinis^70^, and Lab Streaming Layer (LSL) software^71^. The raw EEG and fNIRS data were analysed offline separately. In this study, EEG was used only for determining the eye blink event times and fNIRS was used only for extracting cardiac information. Analysis of brain activity is not included in this paper due to space limitations.

### Pre-processing

The first step after recording the raw EEG data was to pre-process it to segregate the non-brain signals, and preserve as much of the brain signal as possible for further analysis. We pre-processed the EEG data using ICA-based method to separate and remove artefacts from background EEG. As suggested in previous studies, additional steps have to be made prior to ICA decomposition^72,73^. The continuous EEG data was initially epoched into segments based upon the baseline, each Laparoscopic surgery tasks and resting episode, and baseline-corrected using the whole epoch as the baseline^74,75^ . The fragments of data that contain substantial noise with high frequency and high amplitude waves, which could have originated from the gross movements of the participants’ body during EEG recording were deleted. This was done by using a sliding window of 1,000 ms length and removing the fragments in which at least one of the channels exceeded the identified threshold ± 200 µV. Since the EEG data typically have near zero kurtosis values, the electrodes at which the resulting potential values exceed a pre-defined value (e.g. kurtosis value more than 5) were considered as bad channels and removed from the data. The next pre-processing step was band-pass filtering the data using a zero-phase Hamming windowed-sinc FIR filter with passband frequency of 0.16 Hz to 40 Hz to reduce the slow drifts and high frequency artefacts. Afterward, the EEG datasets were down-sampled to 200 Hz to reduce computational and storage cost. In order to segregate components such as eye blinks and eye movements automatically, ADJUST method^76^ implemented in EEGLAB software package^77^, was used. Therefore, the filtered EEG data underwent ICA decomposition using Extended-Infomax algorithm to decompose EEG data into independent components. Then the ADJUST method was applied to detect the independent components associated with eye blink, horizontal and vertical eye movements, and generic discontinuities automatically. The power spectrum, time series and topographic maps of the independent components were also visually inspected by the experimenter. After removing the components associated with artefacts, the pre-processed data was reconstructed for further analysis. In order to estimate the blink rate, we determined the blink times by detecting the sharp peaks in the associated independent components.

In order to capture the cardiac features of interest while minimising other physiological patterns (e.g. Mayer waves) the fNIRS signals were first band-pass filtered in the frequency band 0.5–2 Hz. The signals were converted to hemoglobin concentration changes using the modified Beer–Lambert Law^78,79^.

### Analysis

The channel-averaged oxy-hemoglobin concentration changes contained a strong oscillatory component related to the cardiac pulsation. Accordingly, we assumed that the period of this signal (the interval between every other zero-crossing) was the period of the heartbeat. Next, from the heart rate (reciprocal of the period) the heart rate variability was found. To prepare for the Fourier transform of the HR, we interpolated its values to the regular temporal grid of the original fNIRS signal. We separately considered components in the low-frequency range 0.01–0.15 Hz and the high-frequency range 0.15–0.8 Hz. The latter envelops the frequencies of normal respiration. For every fixed time segment of the HR, the frequency band powers, $LF$ and $HF$, in the low- and high-frequency ranges were computed. The heart rate variability was then calculated as $HRV = {{HF} \mathord{\left/ {\vphantom {{HF} {\left( {HF + LF} \right)}}} \right. \kern-\nulldelimiterspace} {\left( {HF + LF} \right)}}$, thus providing a measure of the extent to which the heart rate was modulated by the respiratory rhythms.

For every participant and experimental episode we calculated the mean, standard deviation, and the maximum and minimum of the HR and HRV. In the case of BR, only the episode mean was used since blinks were insufficiently frequent to allow reliable averaging within shorter time segments. These were used as features for predicting the cognitive load indexed by subjective experience or by performance. Thus, our predictors were the features derived from physiology while the targets of prediction were derived from NASA-TLX reports and behavioural quantities. Note that, following common usage in machine learning, we are using the term prediction to describe a statistical relationship, without implying that the predictor temporally precedes the target.

One of our prediction methods was linear regression (LR). In the simplest case, this involved calculating the correlation between a feature and a target. The result was $r$, the Pearson correlation coefficient, which ranges in the interval $- 1 \le r \le 1$. Using $r$, rather than its square, allowed us to estimate the extent and type (positive or negative) of the association. More generally, multi-variate LR was employed to determine the association between a set of predictors and a target. In such cases the coefficient of determination, $R^{2}$, could be used to indicate the goodness of fit. However $R^{2}$ tends to grow spuriously with the number of predictors. It was therefore preferable to use the adjusted coefficient of determination, $R_{adj}^{2}$, which remains commensurate across different numbers of predictors. We used $R_{adj} = \sqrt {\left| {R_{adj}^{2} } \right|}$ in order to make it approximately comparable with $r$. The absolute value was taken because $R_{adj}^{2}$ is not always positive^80^.

For further generalizability, we deployed an additional prediction method with the same set of predictor and targets. Cascade-forward (CF) artificial neural networks (ANN) with two hidden layers were trained using the Levenberg-Marquard backpropagation algorithm. CF networks are similar to feed-forward (FF) ones but include additional connections from each layer directly to all successive layers. We selected this configuration (hidden layer sizes 10 and 8) after exploring the performance of various FF and CF networks. The performance of the CF-ANN was determined by means of fivefold cross-validation (CV), which trained the network on part of the data (training set) and used it to predict the values of the remaining test set^49^. At each round of CV, 80% of the full data set was selected as the training set. The process was repeated 5 times so that all values had been predicted. The Pearson correlation between the actual and predicted targets was then computed. The fivefold CV itself was repeated a large number of times (we chose 1,000 iterations which appeared more than sufficient for convergence) and the resulting Pearson correlations were averaged. We refer to the resulting average as the cross-validated correlation coefficient, denoted $R_{CV}$. The CF-ANN was intended to capture non-linear dependencies which may be missed by LR and, in addition, help determine the effects of the prediction method on the accuracy. No effort was made to rigorously optimize the network parameters since this was not the main topic of our study. Further validation of $R_{adj}^{{}}$ and $R_{CV}$ is described in Supplementary Information.

In assessing group differences we used the Wilcoxon signed-rank test when the groups contained paired subjects (e.g. subjects that performed the first task v second task) and the Kolmogorov–Smirnov test when they did not (e.g. subjects that performed Ring Transfer v Threading as the first task). These tests were deemed suitable for our study, as they were relatively conservative and did not presuppose the normal distribution. We did not utilise a null hypothesis whose rejection would have required multiple comparisons. The Matlab functions regress, fitlm, cascadeforwardnet, autofindpeaks, cvpartition, corr, kstest2 and signrank were utilized for implementing some of the calculations described above (Matlab R2017b, MathWorks, Inc., Natick, Massachusetts, United States).

The data that support the findings of this study are available on request from the corresponding author.

## Supplementary information

Supplementary Information. (DOCX 1073 kb)
