# Supplementary material for: Physiological correlates of cognitive load in laparoscopic surgery
Source: Sci Rep. 2020 Jul 31;10:12927. doi: 10.1038/s41598-020-69553-3 (PMC7395129; doi:10.1038/s41598-020-69553-3)
Supplement: Supplementary file 1 — Supplementary Information. (DOCX 1073 kb) [file 41598_2020_69553_MOESM1_ESM.docx]

**Physiological Correlates of Cognitive Load in Laparoscopic Surgery**

**Zohreh Zakeri^1, *, +^, Neil Mansfield^1^, Caroline Sunderland^2^, Ahmet Omurtag^1, +^**

^1^Department of Engineering, School of Science and Technology, Nottingham Trent University, Clifton Lane, Nottingham, NG11 8NS, UK

^2^Department of Sport Science, School of Science and Technology, Nottingham Trent University, Clifton Lane, Nottingham, NG11 8NS, UK

*Correspondence: zohreh.zakeri@ntu.ac.uk

^+^These authors contributed equally to this work

**Supplementary Information**.

Fig. S1 depicts the sequence of experimental episodes.

**Supplementary Figure S1.** Experimental procedure.

Fig.S2 shows the training bases (14 cm × 10 cm); Ring stack (Fig S2a) and Threading (Fig S2b) bases that were placed in the LS trainer box to complete each LS task.

**Supplementary Figure S2.** Ring stack base (a), Threading base (b).

In order to investigate the effectiveness of $R_{adj}$and $R_{CV}$, we conducted simulations where a set of target variables $y^{(i)}$ were calculated from predictors $x_{n}^{(i)}$ and a noise term according to the formula , $i=1, ...,N_{D}$. The predictors and noise were random deviates from the standard normal distribution. Size of the data set was $N_{D}=100$, about the same as the data sets in this study. The amplitude of the noise was kept constant $c=1$, while the other coefficients were chosen to generate different types of dependence (no dependence, $a=b=0$; linear dependence, $a=1$, $b=0$; and quadratic dependence, $a=0$, $b=1$) shown in the left, middle and right panels, respectively, of Fig. S3. The simulations were repeated with an increasing number $N_{p}$ of predictor variables, taken as the x-axis in the figure.

In the case of no dependence the goodness of fit, $R$, obtained from the regression model tended to increase with $N_{p}$. This is a well-known artefact of multiple regression ^76^. In our simulations, regardless of the type of dependence, $R\to1$ as $N_{P}\to N_{D}$. The figure indicates that the Pearson coefficient, $r$, of the correlation between the target's actual and predicted values agreed with $R$. When there was only one predictor, $r$ also equalled the absolute value of the correlation between the predictor and the target. As expected, the adjusted value, $R_{adj}$, from regression and the cross-validated value, $R_{CV}$, from the ANN remained small and did not increase with $N_{p}$ when there was no dependence. In the case of linear dependence, all metrics quickly approached unity as the relative importance of the noise decreased with increasing $N_{p}$. With quadratic dependence, however, only $R_{CV}$ remained substantially above zero.


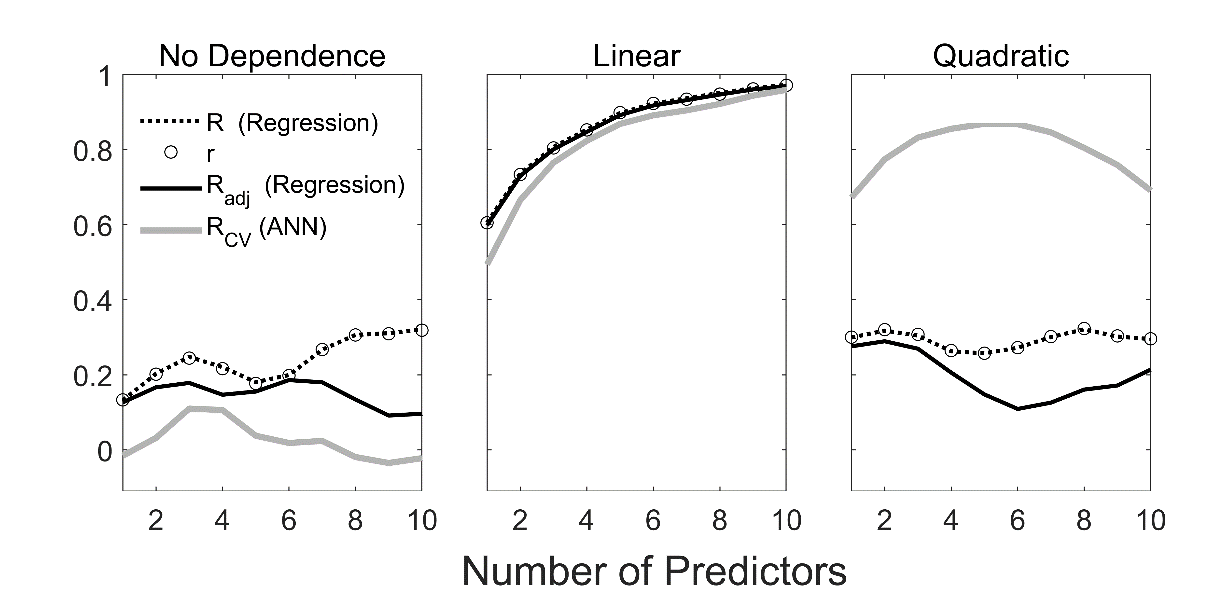


**Supplementary Figure S3.** Indicators of prediction accuracy with simulated data for linear regression and artificial neural network. Each panel represents a different type of dependence of the target on the predictors (no dependence, linear dependence and quadratic dependence).

The results in Supplementary Figure S3, as well as additional simulations that were done with different parameters and more complicated non-linear dependence, suggested that this approach robustly revealed the relationships between the cognitive load and its physiological predictors. Thus, we selected the adjusted-R and cross-validated-R as the metrics for quantifying the results of LR and ANN, respectively.
